# Thyroid dysfunction in preterm infants born before 32 gestational weeks

**DOI:** 10.1186/s12887-019-1792-0

**Published:** 2019-10-29

**Authors:** Hye-Rim Kim, Young Hwa Jung, Chang Won Choi, Hye Rim Chung, Min-Jae Kang, Beyong Il Kim

**Affiliations:** 1 Department of Pediatrics, Bundang CHA Medical Center, CHA University, Seongnam, Republic of Korea; 2 Department of Pediatrics, Seoul National University College of Medicine, Seoul, Republic of Korea; 3 Department of Pediatrics, Seoul National University Bundang Hospital, 82 Gumi-ro 173 Beon-gil, Bundang-gu, Seongnam 13620 Republic of Korea; 4 Department of Pediatrics, Hallym University Sacred Heart Hospital, Anyang, Republic of Korea

**Keywords:** Premature infants, Thyroid dysfunction, Thyroid function test

## Abstract

**Background:**

Thyroid hormones are critical for growth and brain development during the newborn period and infancy. Because of delayed maturation of the hypothalamic-pituitary-thyroid axis in preterm infants, thyroid dysfunction is common, and thyroid stimulating hormone (TSH) elevation is often delayed in preterm infants. The objective of this study was to determine the incidence of thyroid dysfunction requiring levothyroxine treatment and to identify its risk factors in preterm infants.

**Methods:**

A retrospective cohort study was performed on preterm infants who were born before 32 gestational weeks and admitted to a single tertiary academic center for more than 8 weeks between January 2008 and December 2014. In these infants, serial thyroid function tests (TFTs) measuring serum TSH and free thyroxine (fT4) were routinely performed at 1, 3, and 6 weeks of postnatal age.

**Results:**

Of the 220 preterm infants enrolled, 180 infants underwent TFTs at 1, 3, and 6 weeks of postnatal age and were included in the study. Of the 180 infants, 35 infants (19.4%) were started on levothyroxine treatment based on the results of serial TFTs. Among the 35 infants who were treated with levothyroxine, 16 infants (45.7%) had normal results on the initial TFT. Three of these 16 infants continued to have normal results on the second TFT. Thyroid dysfunction requiring levothyroxine treatment was significantly associated with maternal pregnancy-induced hypertension (adjusted odds ratio 2.64, 95% confidence interval 1.02–6.81).

**Conclusions:**

Thyroid dysfunction requiring levothyroxine treatment occurred in nearly one-fifth of preterm infants born before 32 gestational weeks. Nearly half of the preterm infants who were treated with levothyroxine had normal TSH and fT4 levels at 1 week of postnatal age. The findings of the present study suggest that serial TFTs is important to find preterm infants who require levothyroxine treatment.

## Background

Thyroid hormones play a critical role in the maturation of the brain, and hypothyroidism causes neurodevelopmental impairment if not treated properly [1]. Hypothyroidism is common in preterm infants due to immaturity of the hypothalamic-pituitary-thyroid axis, impaired synthesis and metabolism of thyroid hormones, increased demand for thyroid hormone due to nonthyroidal illness, and drug administration [2].

Preterm infants may develop hypothyroidism even when initial thyroid function tests within the first few days of life show normal thyroid-stimulating hormone (TSH) and free thyroxine (fT4) levels. Hypothyroxinemia of prematurity is characterized by low levels of circulating thyroid hormones despite normal TSH levels [3]. Hypothyroxinemia of prematurity is common in preterm infants, occurring in 20% of preterm infants born before 34 gestational weeks and in 29% of very low birth weight (VLBW) infants born before 32 gestational weeks [4, 5], due to blunted postnatal TSH surges and low serum thyroxine (T4) and tri-iodothyronine (T3) levels during the first few weeks of life [2]. Additionally, preterm infants have a higher risk of delayed TSH elevation [6]. Delayed TSH elevation is defined as elevated TSH at the second neonatal screening test despite a normal TSH level at the initial screening test regardless of fT4 levels [7]. The incidence of delayed TSH elevation is estimated to be up to 12% in preterm infants [8, 9], which is significantly higher than that in term infants [10].

Thus, preterm infants are subject to hypothyroxinemia of prematurity and/or delayed TSH elevation [6, 11]. However, whether and how to screen for these thyroid dysfunctions in preterm infants remain controversial. Currently, no universal guidelines are available regarding the indication and timing for screening and the diagnosis and treatment of thyroid dysfunction in preterm infants. Furthermore, whether untreated thyroid dysfunction in preterm infants affects neurodevelopmental outcomes remains unknown.

We aimed to evaluate the incidence of thyroid dysfunction requiring levothyroxine treatment and to identify the associated risk factors in preterm infants born before 32 gestational weeks.

## Methods

### Study subjects

This study enrolled all preterm infants who were born before 32 gestational weeks and admitted to the neonatal intensive care unit (NICU) of Seoul National University Bundang Hospital for more than 8 weeks between January 1, 2008, and December 31, 2014. Since January 2008, serial thyroid function tests (TFTs) measuring both serum TSH and fT4 levels have been performed routinely at 1, 3, and 6 weeks of postnatal age for preterm infants born before 32 gestational weeks and admitted to the NICU. Serum TSH and fT4 levels were measured by in-house radioimmunoassays. To assess changes in serial TFTs over time, we included all infants who underwent complete screening procedure according to the institutional protocol. Even if the results of the initial TFT (from 5 days to 9 days of life) were normal, TFTs were repeated at 3 weeks (from 14 days to 28 days of life) and 6 weeks (from 29 days to 56 days of life) according to our institutional protocol. If the results of the TFT were abnormal (TSH > 6 μU/mL and/or fT4 < 0.8 ng/dL), the TFT was repeated after one or 2 weeks at the discretion of the in-house pediatric endocrinologists (HR Chung and MJ Kang). Levothyroxine treatment was started in infants with serum TSH levels ≥20 μU/mL, regardless of the fT4 level, on any of the serial TFTs or in infants with persistently elevated serum TSH levels (between 10 and 19.9 μU/mL) and persistently low fT4 levels (< 0.8 ng/dL). Levothyroxine treatment was started with an initial dose of 10 to 15 μg/kg depending on the severity of thyroid dysfunction. Follow-up TFTs were performed every 2–4 weeks after starting levothyroxine treatment. Once started, levothyroxine treatment was continued until 2–3 years of age.

### Data collection

Clinical data were retrospectively collected from the electronic medical records. Maternal and perinatal data included gestational diabetes mellitus, pregnancy-induced hypertension (PIH), maternal thyroid disease, premature rupture of membranes (PROM), use of antenatal corticosteroids, gestational age, birth weight, sex, multiple gestations, delivery mode, and Apgar scores at 1 and 5 min. Neonatal morbidity data included respiratory distress syndrome (RDS), patent ductus arteriosus (PDA), necrotizing enterocolitis (NEC), culture-proven sepsis, intraventricular hemorrhage (IVH), cystic periventricular leukomalacia (PVL), retinopathy of prematurity (ROP), and bronchopulmonary dysplasia (BPD). PDA was diagnosed with echocardiography, and symptomatic PDA was defined as hemodynamically significant PDA requiring medical or surgical closure. BPD was defined as the need for supplemental oxygen or assisted ventilation, including nasal continuous positive airway pressure at 36 weeks postmenstrual age (PMA) according to NIH criteria [12]. Early-onset sepsis (EOS) was defined as blood culture-proven bacterial sepsis that occurred before 7 days of life, while late-onset sepsis (LOS) occurred after 7 days of life. Other morbidities included IVH ≥ grade III [13], NEC with modified Bell stage ≥ II [14], and ROP at a high stage requiring laser therapy [15].

### Statistical analysis

Comparisons of continuous variables between the groups were performed with a *Student t*-test for normally distributed variables and a *Mann-Whitney U* test for variables with nonnormal distributions. Categorical variables were compared with a *Pearson chi-square* test. Multiple logistic regression analyses were performed to identify risk factors associated with thyroid dysfunction requiring levothyroxine treatment. Statistical analyses were performed using the IBM SPSS Statistics software package version 24.0 (IBM, Armonk, NY). *P*-values < 0.05 were considered significant.

### Ethical statement

The Seoul National University Bundang Hospital Institutional Review Board (IRB) approved the collection and use of the clinical information of the patients for research purposes before the investigation was started and waived the requirement for informed consent (IRB No. B1806–486-101).

## Results

### Study subjects

From January 2008 to December 2014, 220 preterm infants who were born before 32 gestational weeks and admitted to the NICU for more than 8 weeks were enrolled. Of these 220 infants, 40 infants for whom serial TFTs at three time points (1, 3, and 6 weeks of postnatal age) were not completely performed were excluded. Ultimately, 180 infants who underwent complete screening procedure were included in the analyses. The mean gestational age of the study subjects was 27^+ 5^ weeks (range: 23^+ 1^–31^+ 6^ weeks), and their mean birth weight was 1008 g (range: 420–2275 g). The male to female ratio was 1.1.

### Serial thyroid function tests and levothyroxine treatment

**Fig. 1:**
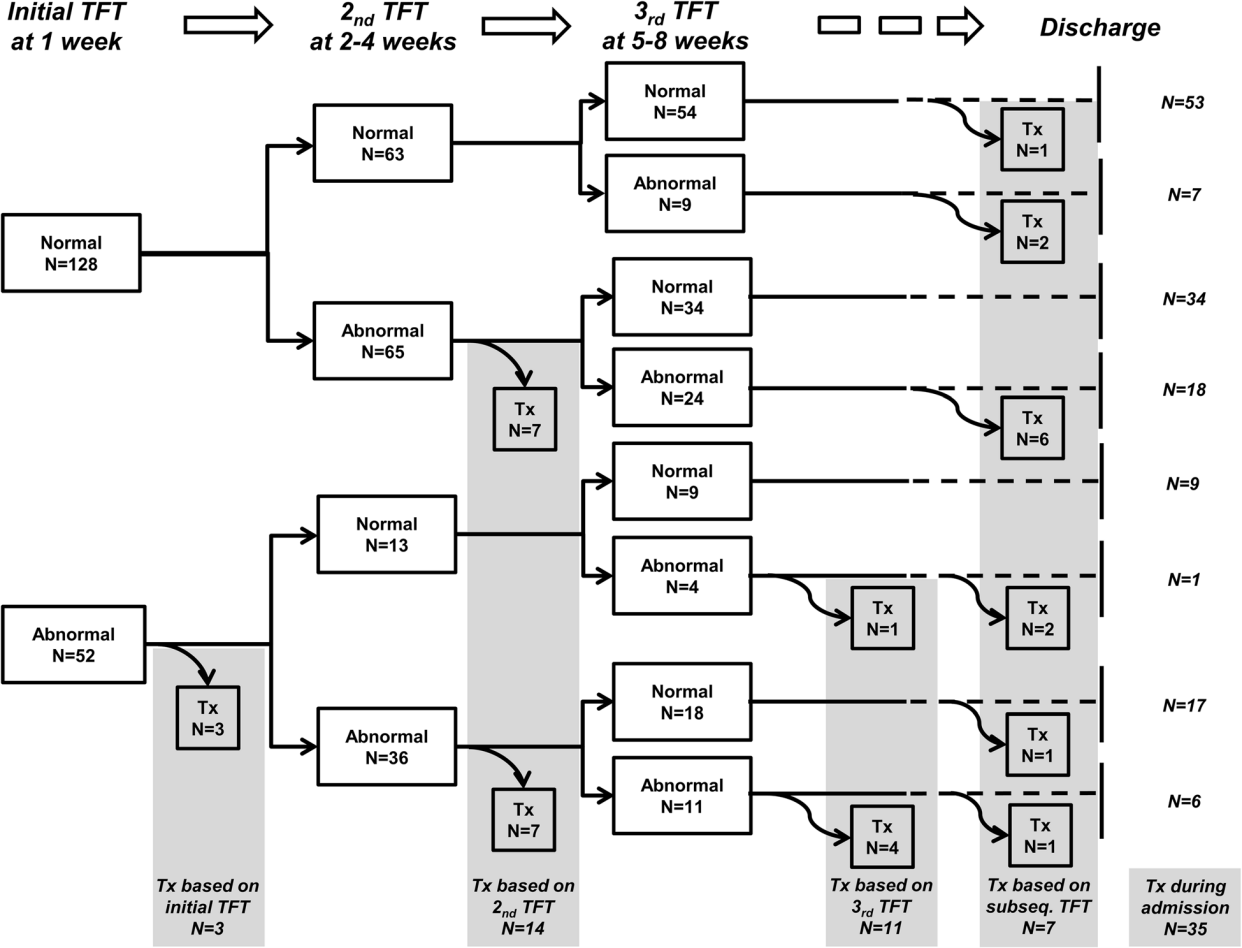
Outcomes of 128 infants with normal initial thyroid function test results and 52 infants with abnormal initial thyroid function test results. TFT, thyroid function test; Tx, levothyroxine treatment; subseq., subsequent

**Fig. 2:**
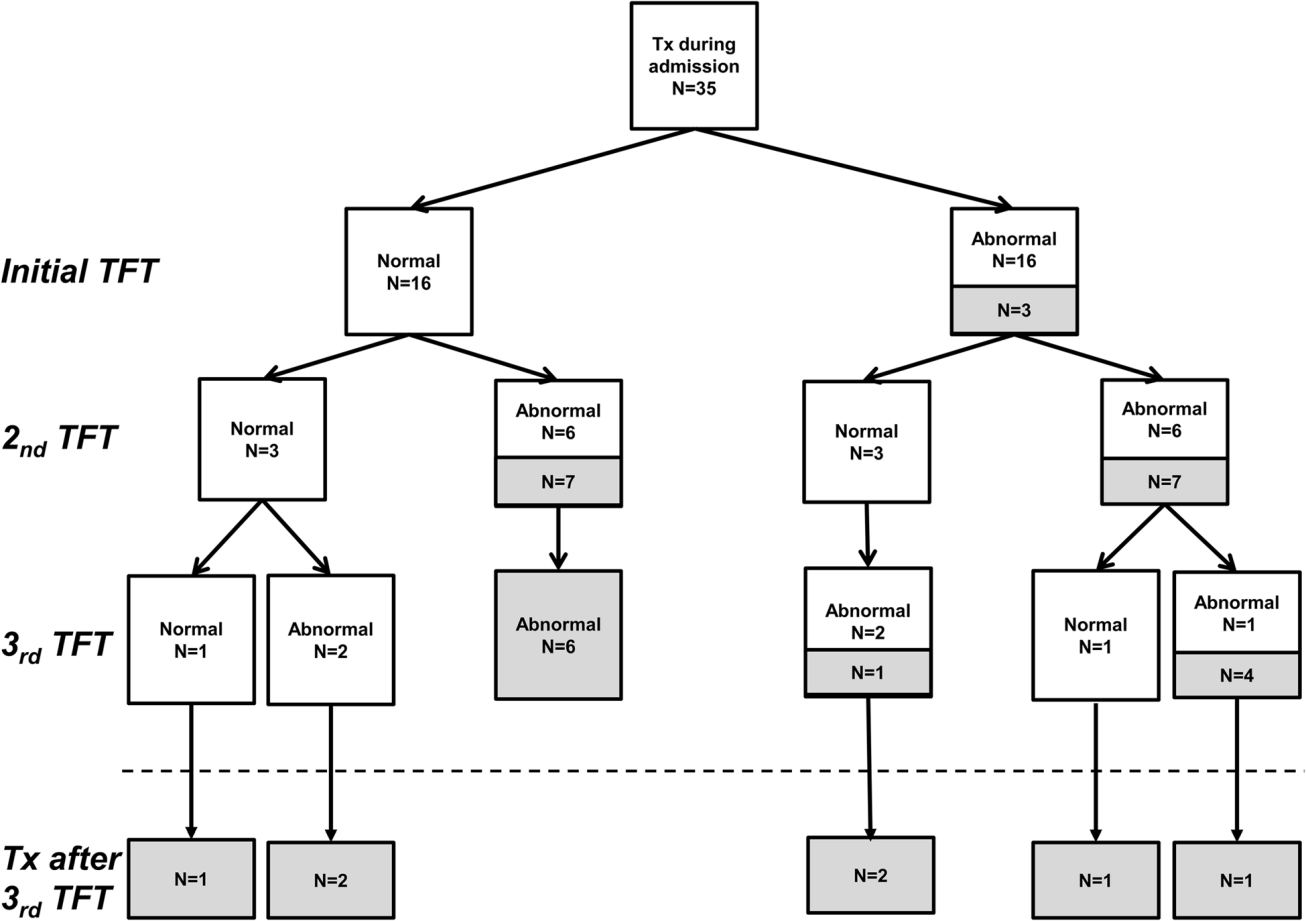
Results of serial thyroid function tests of the 35 infants who were started on levothyroxine treatment during their neonatal intensive care unit admission. TFT, thyroid function test; Tx, levothyroxine treatment. Open boxes indicate infants who were not on levothyroxine treatment at the time of their thyroid function test. Solid boxes indicate infants who were on levothyroxine treatment at the time of their thyroid function test

The mean postnatal ages at the initial, second and third TFTs were 6.9 ± 1.1 (range: 5–9), 22.2 ± 3.7 (range: 15–28), and 42.1 ± 6.6 (range: 29–56) days, respectively. Of the 180 infants, 52 infants (28.9%) showed abnormal results on the initial TFT, and 128 infants (71.1%) showed normal results on the initial TFT. The outcomes of 52 infants with abnormal initial TFT results and 128 infants with normal initial TFT results are presented in Fig. 1. Three infants were started on levothyroxine treatment after exhibiting abnormal results on the initial TFT. Another 14 infants were started on levothyroxine treatment after exhibiting abnormal results on the second TFT, and another 11 infants were started on levothyroxine treatment after exhibiting abnormal results on the third TFT. Finally, 7 infants were started on levothyroxine treatment after exhibiting abnormal results on subsequent TFTs. Consequently, 35 infants (19.4%) were started on levothyroxine treatment during the NICU admission. Of the 35 infants who were started on levothyroxine treatment during the NICU admission, 16 (45.7%) showed normal results on the initial TFT. Of the 16 infants who showed normal results on the initial TFT, 3 (18.8%) continued to show normal results on the second TFT. Of the 3 infants who showed normal results on both the initial and second TFTs, one continued to show normal results on the third TFT. The results of serial TFTs of the 35 infants who were started on levothyroxine treatment during the NICU admission are presented in Fig. 2. Among the 35 infants who were treated with levothyroxine, levothyroxine treatment was started in 13 infants with serum TSH ≥20 μU/mL and normal fT4, 14 infants with serum TSH ≥20 μU/mL and low fT4, and 8 infants with serum TSH levels between 10 and 19.9 μU/mL and low fT4.

The mean serum TSH levels of the infants who were determined not to require levothyroxine treatment according to the initial, second, and third TFTs were 4.61 ± 3.52 μU/mL (*n* = 177), 6.54 ± 4.07 μU/mL (*n* = 163), and 4.46 ± 2.74 μU/mL (*n* = 152), respectively. The mean serum fT4 levels of the infants who were determined not to require levothyroxine treatment according to the initial, second, and third TFTs were 1.29 ± 0.34 ng/dL (*n* = 177), 1.32 ± 0.31 ng/dL (*n* = 163), and 1.34 ± 0.26 ng/dL (*n* = 152), respectively. The mean serum TSH levels of the infants who were determined to require levothyroxine treatment according to the initial, second, and third TFTs were 29.00 ± 35.18 μU/mL (*n* = 3), 80.17 ± 107.49 μU/mL (*n* = 14), and 60.49 ± 71.06 μU/mL (*n* = 11), respectively. The mean serum fT4 levels of the infants who were determined to require levothyroxine treatment according to the initial, second, and third TFTs were 0.69 ± 0.04 ng/dL (*n* = 3), 0.81 ± 0.40 ng/dL (*n* = 14), and 1.01 ± 0.46 ng/dL (*n* = 11), respectively.

### Baseline characteristics of the levothyroxine treatment and nontreatment groups

Thirty-five infants who were started on levothyroxine treatment were compared with 145 infants who were not given levothyroxine treatment. No significant difference in the baseline characteristics was found between the two groups, except that maternal PIH (40.0% vs.16.6%, *p* = 0.002) and cesarean section (82.9% vs. 65.5%, *p* = 0.047) were significantly more common in the levothyroxine treatment group than in the levothyroxine non-treatment group. After adjustment for covariates including gestational age and birth weight percentile as categorical variables (23–25 weeks, 26–28 weeks, 29–31 weeks for gestational age, < 33%, 33–67, > 67% for birth weight percentile), cesarean section, and maternal PIH, only maternal PIH was significantly associated with levothyroxine treatment in multivariable logistic regression analysis (Adjusted odds ratio (OR) 2.64, 95% confidence interval (CI) 1.02–6.81; *p* = 0.045) (Table 1).

**Table 1:** Baseline characteristics of the levothyroxine nontreatment and treatment groups

	Nontreatment *N* = 145	Treatment *N* = 35	*p*	Adjusted p^*^
Maternal age (years)	32.6 ± 3.3	32.1 ± 3.6	0.408	–
Gestational age (weeks^+days^)	27^+ 5^ ± 2^+ 0^	27^+ 6^ ± 2^+ 0^	0.931	–
Birth weight (g)	1026 ± 329	935 ± 266	0.133	–
Male (%)	79 (54.5)	17 (48.6)	0.529	–
Caesarean section (%)	95 (65.5)	29 (82.9)	0.047	–
Multiple gestation (%)	46 (31.7)	6 (17.1)	0.088	–
Antenatal corticosteroids (%)	128 (88.3)	32 (91.4)	0.594	–
Premature rupture of membrane (%)	70 (48.3)	11 (31.4)	0.072	–
Pregnancy-induced hypertension (%)	24 (16.6)	14 (40.0)	0.002	0.045
Gestational diabetes (%)	9 (6.2)	0 (0.0)	0.130	–
Maternal thyroid disease (%)	1 (0.7)	0 (0.0)	1.000	–
1 min Apgar score	4.0 ± 1.9	3.8 ± 1.9	0.586	–
5 min Apgar score	6.1 ± 1.7	5.7 ± 1.7	0.274	–
Respiratory distress syndrome (%)	112 (77.2)	26 (74.3)	0.711	–
Early-onset sepsis (%)	1 (0.7)	2 (5.7)	0.097	–

Values are presented as the means±SDs or numbers (%)

^*^Adjusted for variables including gestational age and birth weight percentile used as categorical variables (23–25 weeks, 26–28 weeks, 29–31 weeks for gestational age; < 33%, 33–67, > 67% for birth weight percentile), cesarean section and maternal pregnancy-induced hypertension

### Neonatal comorbidities in the levothyroxine treatment and nontreatment groups

The incidences of IVH (≥ grade III), symptomatic PDA, NEC (≥ stage II), late-onset neonatal sepsis, BPD, and ROP requiring laser therapy were not significantly different between the levothyroxine treatment group and the levothyroxine nontreatment group. However, cystic PVL was significantly more common in the levothyroxine treatment group than in the levothyroxine nontreatment group (22.9% vs. 9.7%, *p* = 0.044). After adjustment for covariates including gestational age and birth weight percentile as categorical variables (23–25 weeks, 26–28 weeks, 29–31 weeks for gestational age; < 33%, 33–67, > 67% for birth weight percentile), cesarean section, maternal PIH, and cystic PVL, cystic PVL was consistently associated with levothyroxine treatment in multivariable logistic regression analysis (adjusted OR 3.77, 95% CI 1.30–10.96; *p* = 0.015) (Table 2).

**Table 2:** Neonatal comorbidities of the levothyroxine nontreatment and treatment groups

	Nontreatment *N* = 145	Treatment *N* = 35	*p*	Adjusted p^*^
Intraventricular hemorrhage (≥grade III) (%)	7 (4.8)	5 (14.3)	0.059	–
Symptomatic patent ductus arteriosus (%)	81 (55.9)	24 (68.6)	0.187	–
Necrotizing enterocolitis (≥stage II) (%)	13 (9.0)	3 (8.6)	1.000	–
Late-onset sepsis (%)	12 (8.3)	6 (17.1)	0.124	–
Bronchopulmonary dysplasia (%)	73 (50.3)	19 (54.3)	0.710	–
LASER therapy for retinopathy of prematurity (%)	39 (26.9)	11 (31.4)	0.675	–
Periventricular leukomalacia (%)	14 (9.7)	8 (22.9)	0.044	0.015

Values are presented as numbers (%)

^*^Adjusted for variables including gestational age and birth weight percentile used as categorical variables (23–25 weeks, 26–28 weeks, 29–31 weeks for gestational age; < 33%, 33–67, > 67% for birth weight percentile), cesarean section, maternal pregnancy-induced hypertension and periventricular leukomalacia

### Follow-up of the infants with thyroid dysfunction requiring levothyroxine replacement

Of the 35 infants who were started on levothyroxine treatment during the NICU admission, two infants who were transferred to other hospitals were not followed up, and 33 infants underwent regular follow-up examinations at the outpatient clinic in our center after discharge until they were 3 years old. All 33 infants were able to discontinue levothyroxine treatment between 2 and 3 years of age.

## Discussion

In this study, we found that repeated assessment of serum TSH and fT4 levels in preterm infants is necessary to avoid missing thyroid dysfunction requiring levothyroxine treatment. Among the 35 infants who were started on levothyroxine treatment during the NICU admission, 16 (45.7%) had normal results on the initial TFT. Furthermore, 3 of these 16 infants continued to have normal results on the second TFT.

Due to lack of maternal supply of thyroid hormone during the third trimester, preterm infants are vulnerable to develop transient hypothyroxinemia [3]. Additionally, the feedback loop of the hypothalamic-pituitary-thyroid axis is not fully developed, which results in delayed TSH elevation in response to low thyroid hormone levels in preterm infants [16]. Although the thyroid function of preterm infants reaches the same level as that of full-term infants at 4–6 weeks of postnatal age, some may have greater and more persistent delayed TSH elevation or hypothyroxinemia of prematurity, which may require levothyroxine treatment [17]. In the present study, the overall incidence of thyroid dysfunction requiring levothyroxine treatment was 19.4% in preterm infants who were born before 32 gestational weeks and hospitalized more than 8 weeks. In recent studies, the incidence rates of thyroid dysfunction requiring levothyroxine treatment were 12.2% in VLBW infants and 9.1% in preterm infants born before 30 gestational weeks [18, 19]. Compared to these recent studies, our study found a higher incidence of thyroid dysfunction requiring levothyroxine treatment. Because only preterm infants who were hospitalized for more than 8 weeks were enrolled in our study, the study population might have consisted of more immature and sicker preterm infants. However, considering the differences in study populations, the incidence of thyroid dysfunction requiring levothyroxine treatment is notable. Therefore, some researchers have suggested that TFTs should be repeated at regular intervals to identify thyroid dysfunction associated with delayed TSH elevation or hypothyroxinemia of prematurity in preterm infants. Different approaches have been suggested to avoid missing thyroid dysfunction requiring levothyroxine treatment. Some guidelines recommend routine screening tests for thyroid function at 2 and 6 weeks of age in all VLBW and LBW infants in the NICU [20]. In some regions of the United States, serum fT4 and TSH levels are measured at 2, 6 and 10 weeks of postnatal age in all VLBW infants [21]. The European Society for Pediatric Endocrinology guidelines recommend that a repeat specimen should be collected at 2 weeks of postnatal age or 2 weeks after the first screening tests for thyroid function for preterm infants or VLBW infants [22]. In our center, both serum fT4 and TSH levels are measured at 1, 3 and 6 weeks of postnatal age for preterm infants born before 32 gestational weeks, as reported previously [8]. In the present study, the rates of abnormal results on the initial, second, and third TFT were 28.9% (52/180), 57.1% (101/177), and 29.4% (48/163), respectively. Of the 128 infants who showed normal results on the initial TFT, 65 (50.8%) showed abnormal results on the second TFT. Furthermore, 9 (14.3%) of the 63 infants who had normal results consecutively on the initial and second TFTs showed abnormal results on the third TFT. These results suggest that a single or two TFTs may be insufficient to identify thyroid dysfunction in preterm infants. In our study, only one infant was further started on levothyroxine treatment after showing normal results on all three serial TFTs. This infant had severe encephalomalacia. Subsequent TFTs were performed on this infant after 8 weeks of age because of clinical suspicion of thyroid dysfunction.

Thyroid dysfunction is common in more immature preterm infants. The incidence of hypothyroxinemia of prematurity is inversely correlated with gestational age [23]. Van Wassenaer et al. [2] found that smaller and more immature preterm infants have significantly lower fT4 levels than more mature preterm infants or term infants. Delayed TSH elevation has also been associated with low birth weight [18, 19]. In contrast, thyroid dysfunction requiring levothyroxine treatment was not associated with gestational age or birth weight in this study. A few studies have demonstrated that delayed TSH elevation is not associated with gestational age or birth weight [24]. These inconsistent results among studies may be explained by differences in sample sizes, reference ranges for TFTs or definitions of hypothyroxinemia of prematurity and delayed TSH elevation between the studies.

Infants who required levothyroxine treatment were delivered by cesarean section more frequently in the present study. Several studies demonstrated that the association between cesarean section and delayed TSH elevation [7, 25, 26]. However, the association of cesarean section with the levothyroxine treatment might be due to a confounding effect resulting from an association between maternal PIH and cesarean section. Because maternal PIH was a common indication for preterm cesarean section, cesarean section was significantly associated with maternal PIH (*p* = 0.007).

After adjustment for covariates, only maternal PIH was significantly associated with thyroid dysfunction requiring levothyroxine treatment on multivariate analysis in this study. Similar to our results, several studies reported that higher TSH levels or lower fT4 levels were observed in preterm infants born to mothers with PIH [27–29]. Because maternal PIH can lead to placental insufficiency, it may decrease placental passage of T4 from the mother to fetus, and therefore, hypothyroxinemia or delayed TSH elevation after preterm birth might be aggravated.

Among the comorbidities examined, only cystic PVL was significantly associated with thyroid dysfunction requiring levothyroxine treatment. Thyroid hormones play an important role in the development of the central nervous system during the fetal and early neonatal periods [30]. In particular, thyroid hormones are very important for the development of cerebral circumvolutions and white matter and myelination [31]. Hypothyroxinemia of prematurity has been associated with PVL [32, 33]. In a retrospective multicenter study of VLBW infants, cerebral white matter injury was twice as common in infants exposed to low total T4 levels compared to infants exposed to higher total T4 levels [32]. The causal relationship between thyroid dysfunction requiring levothyroxine and cystic PVL could not be determined in this study. However, some speculations can be made on this association. First, exposure to hypothyroxinemia before initiation of levothyroxine treatment might have contributed to the development of white matter injury. Second, thyroid dysfunction requiring levothyroxine treatment might have shared risk factors or underlying conditions with cystic PVL.

We also found that all preterm infants with thyroid dysfunction requiring levothyroxine treatment had transient hypothyroidism, suggesting that thyroid dysfunction in preterm infants may often be transient. Recent studies have advocated that the course of hypothyroidism is transient in many preterm infants and that discontinuing levothyroxine supplementation is possible before 3 years of age [34, 35]. Preterm birth and associated clinical conditions can result in temporary thyroid dysfunction that can persist for several days to months [36]. However, long term neurodevelopmental outcomes of transient hypothyroidism should be evaluated.

Our study has some limitations. The retrospective design involving a single center and a lack of long-term neurodevelopmental outcome data are weak points of the present study. Moreover, preterm infants who were born before 32 gestational weeks and admitted to the NICU for more than 8 weeks were included in the study population. These infants would have been more immature and sicker and, thus, would have been more likely to exhibit higher incidence of severe ROP, PVL, and thyroid dysfunction requiring levothyroxine treatment. Further studies are required to determine the long-term neurodevelopmental consequences of thyroid dysfunction requiring levothyroxine treatment and to identify optimal thyroid function screening methods for preterm infants.

## Conclusion

Thyroid dysfunction requiring levothyroxine treatment occurred in nearly one-fifth of preterm infants born before 32 gestational weeks. Nearly half of the preterm infants who were treated with levothyroxine had normal TSH and fT4 levels at 1 week of postnatal age. The findings of the present study suggest the importance of serial TFTs in preterm infants to identify patients who require thyroid replacement therapy.

## Supplementary information

**Additional file 1.** The raw data set including demographics and all results of serial thyroid function tests of the study population.

## Data Availability

The dataset supporting the conclusions of this article is included within the article and its Additional file 1.

## Abbreviations

BPD: Bronchopulmonary dysplasia
EOS: Early-onset sepsis
fT4: Free thyroxine
IVH: Intraventricular hemorrhage
LBW: Low birth weight
LOS: Late-onset sepsis
NEC: Necrotizing enterocolitis
NICU: Neonatal intensive care unit
PDA: Patent ductus arteriosus
PIH: Pregnancy induced hypertension
PMA: Postmenstrual age
PROM: Premature rupture of membrane
PVL: Periventricular leukomalacia
RDS: Respiratory distress syndrome
ROP: Retinopathy of prematurity
T3: Tri-iodothyroxine
T4: Thyroxine
TFT: Thyroid function test
TSH: Thyroid stimulating hormone
VLBW: Very low birth weight
